# Wet-electrospun porous freeform scaffold enhances colonisation of cells

**DOI:** 10.1016/j.mtbio.2025.101997

**Published:** 2025-06-16

**Authors:** Haoyu Wang, Xueying Xu, Yifei Qin, Hongyi Chen, Yuexin Wang, Joel Turner, Jianping Zhang, Maryam Tamaddom, Feng-Lei Zhou, Gareth Williams, Chaozong Liu

**Affiliations:** a Division of Surgery & Interventional Science, University College London, Royal National Orthopaedic Hospital, Stanmore, HA7 4LP, UK; b Department of Computer Science, University College London, London, NW1 2AE, UK; c Department of Mechanical Engineering, University College London, London, WC1E 7JE, UK; d UCL School of Pharmacy, University College London, 29–39 Brunswick Square, London, WC1N 1AX, UK; e Division of Surgery and Interventional Sciences, University College London, Royal Free Hospital, Pond St, London, NW3 2QG, UK; f Centre for Medical Image Computing, Department of Medical Physics and Biomedical Engineering, University College London, London, WC1V 6LJ, UK; g College of Textiles and Clothing, Qingdao University, Qingdao, 266071, China

**Keywords:** Polycaprolactone (PCL), Wet-electrospinning, 3D porous scaffold, Cell infiltration, Gelatin conjugation, ECM deposition

## Abstract

Osteoarthritis is a degenerative disease characterized by the progressive deterioration of articular cartilage. Electrospun scaffolds have shown promise in the regeneration of degraded areas due to their highly interconnected and extracellular matrix-mimicking structures. However, current electrospun scaffold-based therapies are limited by the constraints of 2D cell culture. In this study, a novel wet-electrospinning technique to generate polycaprolactone (PCL) porous 3D scaffolds was developed. The wet-electrospun yarns were collected via vortex, allowing for loosely interconnected yarns, thereby enhancing cell infiltration. Sodium hydroxide (NaOH) treatment was used to introduce carboxyl groups on PCL fibres, followed by gelatin conjugation via N-hydroxysuccinimide (NHS) and 1-ethyl-3-(3-dimethylaminopropyl) carbodiimide (EDC) crosslinking. Comparative analysis between conventional electrospun 2D dense and wet-electrospun 3D porous scaffolds revealed significant advantages in porosity, reaching up to 99.5 % in the 3D matrices. Subsequent in vitro evaluations demonstrated full-thickness cell infiltration in the 3D high-porosity scaffold after 7 days, as confirmed by SEM and confocal images. Further analysis on day 14 revealed the deposition of glycosaminoglycans (GAGs) and collagen. This research introduces a novel technique for fabricating high-porosity scaffolds that facilitate full-thickness 3D cell culture. These novel high-porosity, gelatin-conjugated scaffolds enhance cell colonisation and deposition. Overall, these high-porosity scaffolds overcome the limitations of conventional electrospinning, enabling 3D cell culture and offering new opportunities for cartilage regeneration and reconstruction.

## Highlights

• A novel wet-electrospun PCL 3D porous scaffold with interconnected loosened yarn achieves 99.5 % porosity.
• Gelatin-conjugated porous scaffold allows cells' full-thickness infiltration by Day 7.
• Scaffold remodeling and ECM deposition were observed in the 400 μm deep zone of the porous scaffold.

## Graphical abstract

**Figure:**
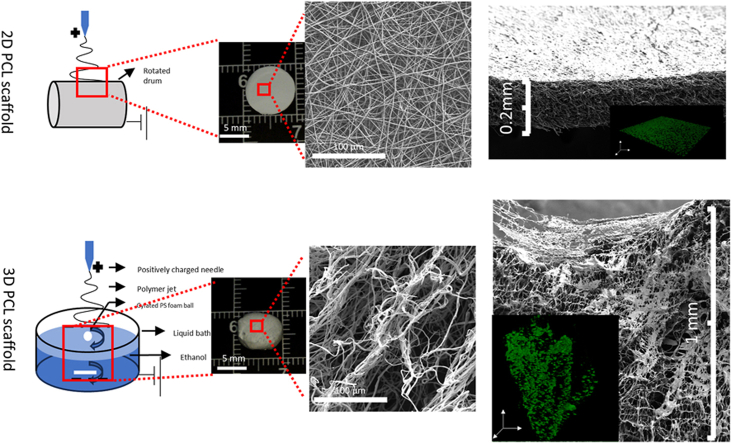

## 1 Introduction

Osteoarthritis (OA) affects approximately 600 million people globally, significantly contributing to years lived with disability, reduced wellbeing, and increased psychological distress [[1], [2], [3], [4]]. The molecular mechanisms of OA remain debated. Potential triggers include long-term stress concentration from improper force line alignment, acute injuries, subchondral bone defects, and a combination of genetic and immune factors [[5], [6], [7], [8]]. OA progression is marked by defects and inflammation in the superficial cartilage layer, characterized by low cell density and no vascular supply, leading to irreversible damage without intervention [8].

Scaffold-based therapy has emerged as a promising approach for cartilage regeneration due to its ability to provide a tunable microenvironment for tissue reconstruction [8,9]. Among these, electrospinning techniques are gaining attention for their ability to produce ECM-mimicking fibrous structures and interconnected networks [10,11]. However, traditional electrospinning methods result in high-density fibre deposition, leading to small pore sizes that hinder cell infiltration, creating a predominantly 2D culture environment unsuitable for 3D tissue reconstruction such as articular cartilage [12].

A commonly observed partially swollen and bonded density fibre structure is attributed to an improper evaporation rate, causing a fixed and locked fibrous structure. [13,14]. Several approaches have been proposed to modify fibre deposition, including the use of sacrificial materials such as sodium chloride [15], PEO fibres [16] and PEG microparticles [17]. However, these methods failed to introduce interconnected channels for cell migration.

Wet-electrospinning has emerged as a promising alternative. In this technique, fibres are deposited into a liquid bath, preventing fibre adhesion and allowing dynamic liquid flow to rearrange fibres into specific structures or form yarn [18,19]. For instance, Wu et al. introduced dynamic liquid flow through a two-layer liquid bath with a hole in the upper layer, producing twisted yarns through the dynamic flow, which were then collected for woven scaffold fabrication [20]. The fabrication of a porous 3D scaffold via dynamic wet-electrospinning remains challenging, owing to unidirectional or vortex flows that drag the fibre and lead to structural collapse.

In wet-electrospinning, polycaprolactone (PCL) is a widely used material due to its biocompatibility, ductility, and spinnability [21,22]. However, its hydrophobic nature hinders cell adhesion [23]. Gelatin, a bioactive material derived from the hydrolysis of collagen and containing the RGD sequence for cell recognition, promotes cell adhesion and ECM deposition [[24], [25], [26]]. Combining PCL with gelatin typically involves the binding of carboxyl groups to amine groups, with common commercial crosslinking agents like NHS and EDC facilitating this process [27,28]. NaOH treatment is often used to slowly hydrolyse PCL [29], exposing more carboxyl groups for binding.

In this study, an improved wet-electrospinning technique was developed by introducing a floating, gyrated polystyrene (PS) ball-shaped collector. The dynamic liquid flow generated by a magnetic stir bar directed the deposited yarns into the PS ball, creating a PCL high-porosity scaffold consisting of interconnected loosened yarns. This 3D porous scaffold was conjugated with gelatin on surface carboxyl groups utilizing 1-ethyl-3-(3-dimethylaminopropyl) carbodiimide (EDC) and N-hydroxysuccinimide (NHS) crosslinking techniques, resulting in improved cell viability and proliferation. This gelatin-conjugated 3D porous scaffold showed full-thickness cell infiltration and enhanced ECM deposition, indicating its potential application in cartilage regeneration.

## 2 Materials and methods

### 2.1 Materials

Polycaprolactone (PCL, Mw = 80,000), N,N-dimethylformamide (DMF), chloroform, ethanol, sodium hydroxide (NaOH), cold water fish gelatin, N-hydroxysuccinimide (NHS), N-(3-dimethylaminopropyl)-N′-ethylcarbodiimide hydrochloride (EDC), 70 % perchloric acid, 2-(N-morpholino) ethane sulfonic acid buffer (MES buffer, pH 4.7), phosphate-buffered saline (PBS, pH 7.2), Toluidine blue (TBO), Safranin O, sodium chloride (NaCl), chloramine T, dimethylaminobenzaldehyde (DMBA), and hydroxyproline were procured from Sigma (Merck, UK). PrestoBlue™, Live/Dead kit, Phalloidin, 4′,6-diamidino-2-phenylindole (DAPI), and OCT mounting medium were obtained from Thermo Fisher. GAGs assay kits were purchased from Biocolor. All materials were used as received, without further purification.

### 2.2 Fabrication of 2D dense and 3D porous wet-electrospun scaffold

PCL was dissolved in a solvent mixture of DMF/chloroform (2/8 v/v) at a concentration of 13 wt%. The solutions were stirred overnight at room temperature to ensure homogeneity. Electrospinning was conducted with the following parameters: a 15 cm distance between the needle and collector, a flow rate of 2.5 ml/h, and an applied voltage of 13 kV. All samples were stored in a vacuum desiccator for 14 days before testing.

**Fig. 1:**
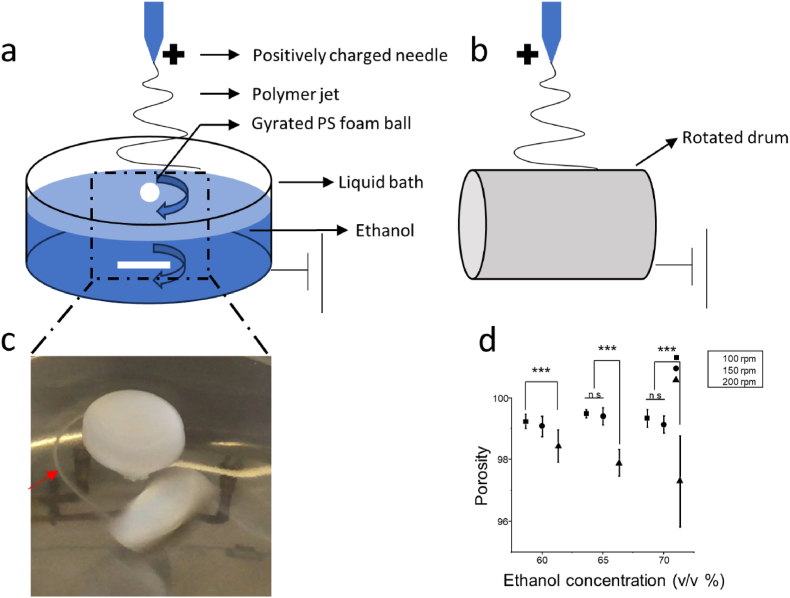
Schematic setups of fabrication: a) Improved wet-electrospun set-up created for the fabrication of 3D porous scaffold, and b) Conventional rotating electrospinning used for 2D membrane fabrication. c) Photograph capturing the process of 3D matrix fabrication. d) The relationship between porosity and processing parameters, including the rotation speed of the magnetic stir bar and the concentration of ethanol.

The fabrication of 3D porous and 2D dense scaffolds is depicted in Fig. 1a and b. For 2D scaffold fabrication, fibres were collected on a rotating drum with a 10 cm diameter, rotating at a speed of 80 rpm. The drum was mounted on a single-axis reciprocating platform, moving at a speed of 1 cm/s with a 10-s cycle. Fibrous membranes with dimensions of approximately 10 cm × 30 cm × 200 μm were generated and cut into 1 cm disk shapes.

The 3D porous wet-electrospun scaffolds were fabricated using an improved wet-electrospinning set-up. A 15 cm diameter cylindrical stainless steel liquid bath served as the collector, grounded during electrospinning and filled with ethanol at concentrations ranging from 60 to 70 v/v%, which allowed fibres to wet but not sink to the bottom. Within the liquid bath, a polystyrene (PS) foam ball gyrated, collecting fibres. This PS ball floated on the surface, its gyration driven by the dynamic liquid flow.

Once electrospun, the non-woven fibre net was deposited into the liquid bath, following the dynamic flow rearrangement into yarns (marked by a red arrow in Fig. 1c), and entangling with the ball, forming a highly porous structure. Specifically, once a high voltage was applied to the needle tip, the polymer jet elongated and solidified, depositing a non-woven fibre net on the ethanol's surface. The ethanol then wetted the fibre net, causing the fibres to deform and rearrange, following the dynamic flow towards the bath's centre where a small vortex held the PS ball. This rearrangement process transformed the non-woven fibre net into yarn. Due to the processing within the liquid bath, the yarn maintained its loose structure, reaching 99.5 % porosity (Fig. 1d).

This entanglement process, driven by the dynamic liquid flow (controlled by a magnetic stir bar at speeds of 100, 150, and 200 rpm), resulted in a high-porosity 3D bulk which was then cryo-cut into dish shapes with Φ = 1 cm and 2 mm thickness. Morphological characteristics were determined using scanning electron microscopy (SEM, Phenom ProX, USA). Samples were coated with gold and imaged at a 5 kV acceleration voltage. SEM images were subsequently analysed using ImageJ 1.8.0 (NIH, USA) to compute the mean fibre diameter and pore size. (n = 100). For fibre diameter measurement, 100 fibres were randomly selected and measured. The SEM images of the scaffolds and optical images of trypsinised cells or cells seeded on the culture plate were thresholded and analysed to quantify their size.

### 2.3 Surface modification

PCL 2D and 3D scaffolds were immersed in a 1 M NaOH solution maintained at 25 °C. The immersion durations assessed were 2, 4, 6, 8, 10, and 12 h. For the evaluation of electrospun PCL surface functional group changes, the density of the carboxyl groups on the surface was qualitatively assessed using TBO (n = 5). Samples were immersed in a toluidine blue solvent (comprising 0.01 M NaOH, 0.2 wt% NaCl, and 0.04 wt% TBO) for 4 h. After rinsing thrice with distilled water, the samples were submerged in 70 % perchloric acid to detach TBO [[30], [31], [32]]. The TBO supernatant (150 μl) was then extracted and its absorbance was measured against a standard curve. Spectral changes were recorded using Total Reflectance Fourier Transform Infrared Spectroscopy (FTIR, PerkinElmer Spectrum 100, USA) in the range between 400 cm^−1^ to 4000 cm^−1^. The range between 1000 cm^−1^ to 1400 cm^−1^ and 3400 cm^−1^ to 3700 cm^−1^ were analysed. The crystallization index (CI) was calculated as the ratio between 1294 cm^−1^ and 1165 cm^−1^ to indicate the PCL crystallization changes. The water contact angle was determined using a Contact Angle Goniometer (Ossila, UK). A 10 μl drop of deionized water was placed on the sample, and its shape was captured at 1, 3, 5, 10, 20, 30, and 60 s (n = 5).

For mechanical property evaluation, 2D scaffolds, sized 30 × 4 × 0.2 mm, were subjected to tensile testing (n = 13). The test was conducted referencing ISO 527-1 with 20 mm initial gap, 15 kPa pre-load stress, and a test speed of 50 mm/min (Zwick 005, ZwickRoell, Germany).

Morphological changes at 4 h and 8 h timepoints and fibre diameter variations were determined using scanning electron microscopy (SEM, Phenom ProX, USA) as described before. Thermal changes were gauged using differential scanning calorimetry (Mettler Toledo, USA). A heating rate of 10°/min was set, with a temperature range of 25–80 °C. Samples weighed approximately 20 mg and preheated to remove thermal history. The crystallinity (X) of the samples was computed using the formula: X = h/H, where 'h' represents the heat of fusion normalized to the sample's mass, and 'H' denotes the heat of fusion for 100 % crystalline PCL (136 J/g) [33]. The melting temperature for the samples was identified at the peak.

### 2.4 Gelatin conjugation

For gelatin conjugation, the 2D and 3D samples were first activated the carboxyl groups via NHS/EDC activation buffer (comprising 2 mM EDC, 5 mM NHS, and MES buffer at pH 4.7) for 30 min at room temperature. Following activation, the samples were rinsed three times with phosphate-buffered saline (PBS). They were then submerged in a 1 wt% gelatin solution for 2 h, followed by another three washes with PBS to remove any unconjugated gelatin.

The density of conjugated gelatin in relation to the NaOH treatment duration, and its degradation profile were assessed (n = 5). Gelatin density was determined using Bergman and Loxley method [34,35]. Specifically, hydroxyproline present in gelatin was oxidized with chloramine T and subsequently coupled with DMBA. This reaction yields a coloured product, the absorbance of which was measured at 550 nm. The resulting values were compared against standard curves for quantification.

Samples optimized with the NaOH treatment (1 M, 37 °C, 4 h) were subjected to gelatin conjugation, and further analyses were conducted using FTIR, X-ray photoelectron spectroscopy (XPS), and cellular assays. Functional group changes post-conjugation were analysed using FTIR and XPS. FTIR tests were conducted as previously described, and the range between 1450 and 1700 cm^−1^ for amide bands and between 3400 and 3700 cm^−1^ for OH stretching was analysed. For XPS analysis, the O1s, C1s, and overall spectra were recorded using X-ray photoelectron spectroscopy (XPS, Kratos Axis Supra).

### 2.5 In vitro evaluation

Bone marrow mesenchymal stem cells (BMSCs) from sheep, between passages 5 and 10, were utilized for in vitro evaluations, including viability, infiltration, and ECM deposition tests. The culture of BMSCs was described in a previous study [36]. In brief, MSCs were cultured at 37 °C and 5 % CO_2_, with the medium changed every two days (Dulbecco's Modified Eagle Medium, Sigma, Merck, UK; 10 % fetal calf serum, First Link, UK; and 1 % penicillin and streptomycin, Gibco, UK).

A total of 60,000 MSCs were seeded into each well of a 24-well plate. Cell morphology after 24 h was visualized using Phalloidin/DAPI staining and observed via SEM. Samples were fixed with 2.5 % glutaraldehyde for 30 min, dehydrated with a graded ethanol series from 10, 20, 30, 40, 50, 60, 70, 80, 90, 95, to 100 v/v%, and air-dried overnight. Phalloidin targeted F-actin and DAPI stained the nuclei. For phalloidin staining, cells were permeabilized with 0.1 % Triton X-100 for 10 min, followed by incubation with phalloidin for 30 min at room temperature. DAPI staining was then performed by incubating the samples with DAPI solution for 5 min to stain the nuclei. The stained samples were then observed using a fluorescence microscope (Zeiss, Axioscope, Germany).

For viability tests, PrestoBlue™ was used to quantify BMSCs on days 1, 3, and 7, comparing the PCL 2D and 3D scaffolds (n = 5). The procedure involved adding 10 % (v/v) PrestoBlue™ reagent to the culture medium and incubating the cells at 37 °C for 2 h. After incubation, the fluorescence was measured at an excitation wavelength of 560 nm and an emission wavelength of 590 nm using a plate reader. Cell viability was further assessed using the Live/Dead kit. Scaffolds seeded with BMSCs on days 3 and 7 were incubated with a solution containing 4 μM Ethidium Homodimer-1 (for dead cells) and 2 μM Calcein AM (for live cells) dissolved in PBS for 30 min at room temperature. The stained cells were then visualized under the fluorescence microscope to differentiate live (green) and dead (red) cells.

Infiltration was evaluated by observing the cross-sectional areas using histological analyses, SEM imaging, and confocal microscopy. Cell-seeded samples from days 3, 7, and 14 were embedded in an OCT mounting medium, cryosectioned (25 μm, Leica CM1860), and stained with DAPI to determine infiltration depth. For the 7-day cell culture on both the 2D membrane and 3D matrix, samples were fixed as previously described. Post-fixation, samples were subjected to cryofracture in liquid nitrogen to obtain fractured 3D blocks for exposing cross-sectional areas under SEM observation. Given their low mechanical strength, samples were freeze-dried for 48 h to prevent collapse due to surface tension during drying. These samples were then gold-coated and examined under SEM. The samples were also stained with Calcein AM and scanned with a confocal microscope (Leica SP8, Leica Microsystems, Germany). Samples were cultured with Calcein AM for 30 min at room temperature, fixed as described before, and scanned under the confocal microscope. The z-stack images were automatically generated via the software.

ECM deposition was evaluated by analysing collagen and glycosaminoglycans (GAGs) (n = 3). Collagen content was determined using the hydroxyproline concentration test as described previously. The collagen content was calculated based on the assumption that hydroxyproline constitutes approximately 13.5 % of the total collagen [37,38]. Gelatin-conjugated scaffolds without cell seeding were used as controls to account for pre-existing hydroxyproline content from gelatin. Their hydroxyproline levels were subtracted from the experimental groups to quantify newly deposited ECM. GAG deposition was quantified with a GAG assay kit. Samples were digested with papain solution at 60 °C for 24 h, then mixed with dimethylmethylene blue dye reagent. Absorbance was measured at 525 nm and compared against chondroitin sulfate standards. Additionally, Safranin O staining was performed to visualize GAGs. Samples were fixed in 4 % paraformaldehyde for 30 min, washed with PBS, and stained with 0.1 % Safranin O solution for 5 min. Excess stain was removed by washing with acetic acid, and stained samples were imaged under a light microscope to assess GAG distribution.

### 2.6 Statistical analysis

The results were presented as mean ± standard deviation. Statistical analyses and plotting were conducted using Origin software (OriginLab Corporation, USA). For the statistical analysis of characterisation results, either a *t*-test or a Mann-Whitney test was applied, depending on the results of the D'Agostino-Pearson normality test. For in vitro evaluation, two-way analysis of variance (ANOVA) was used to analyse the significance level of differences between different groups, with results displayed as P-values, where ∗, ∗∗, and ∗∗∗ correspond to P < 0.05, P < 0.01, and P < 0.005, respectively.

## 3 Results

### 3.1 2D dense scaffold vs 3D porous scaffolds

**Fig. 2:**
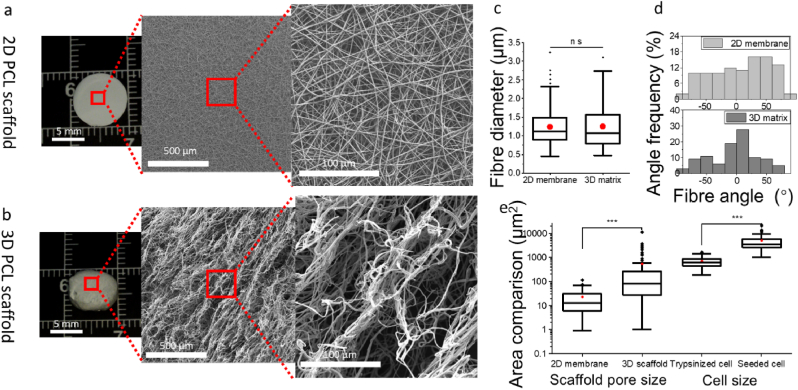
Comparison of 2D and 3D scaffolds. a) Photographic representation of a conventional 2D electrospun PCL dense scaffold, and b) a 3D freeform wet-electrospun porous scaffold, along with their corresponding SEM images at both low and high magnifications; c) fibre diameter; d) fibre orientation; e) comparison of scaffold pore area and cell size. Cell size was measured post-trypsinisation and one day after seeding on tissue culture plates (TCP). Statistical significance is indicated by P-values, where ∗, ∗∗, and ∗∗∗ correspond to P < 0.05, P < 0.01, and P < 0.005, respectively.

The PCL 2D and 3D scaffolds (Fig. 2a and b), fabricated via conventional electrospinning and freeform wet-electrospinning, were visually examined under an optical lens, and their structures and morphologies were compared using SEM. The conventional electrospun samples displayed a 2D flat dense membrane appearance, while the wet-electrospun porous samples exhibited a 3D shape. All samples were cryo-punched into 1 cm disks for further examination.

In the wet-electrospinning process, fibres were rearranged into yarns, as described previously (Fig. 1c). These yarns maintained a loose structure with interconnected curly fibres binding together to form a three-level structure: individual curly fibres, loose yarn consisting of these curly fibres, and the overall 3D porous scaffold made up of the loose yarn (Fig. 2b).

Fibres in both 2D and 3D scaffolds were smooth with average fibre diameters around 1.25 μm, showing no significant difference (Fig. 2c). However, the rearrangement process made the fibres more oriented (Fig. 2d), which could be attributed to higher dynamic fluid velocity arising from magnetic stir bars. The fibre rearrangement in the water bath led to larger pores in the scaffold structure compared with the conventional electrospun fibres, which could allow cell infiltration (Fig. 2e).

For example, in the 2D flat samples, fibres were tightly deposited, displaying an average pore size of 22.72 ± 24.21 μm^2^. This is considerably smaller than the trypsinised BMSCs cell size of 579.94 ± 236.57 μm^2^ and the BMSCs cells seeded on the tissue culture plate, which measured 5045.26 ± 4201.84 μm^2^. In contrast, the interconnected loose yarn structure in the 3D scaffold, as observed under SEM, showed small pores from curly fibres, medium pores between these curly fibres forming a yarn-like structure, and large pores between these yarn structures. These large pores are represented in the box chart (Fig. 2e) as outliers, whose range matched that of post-trypsinisation and adhesive cells. Therefore, the 3D porous scaffold is likely to allow cells to seed and attach inside the 3D scaffold.

### 3.2 Carboxyl group introduction optimisation via NaOH solution process

**Fig. 3:**
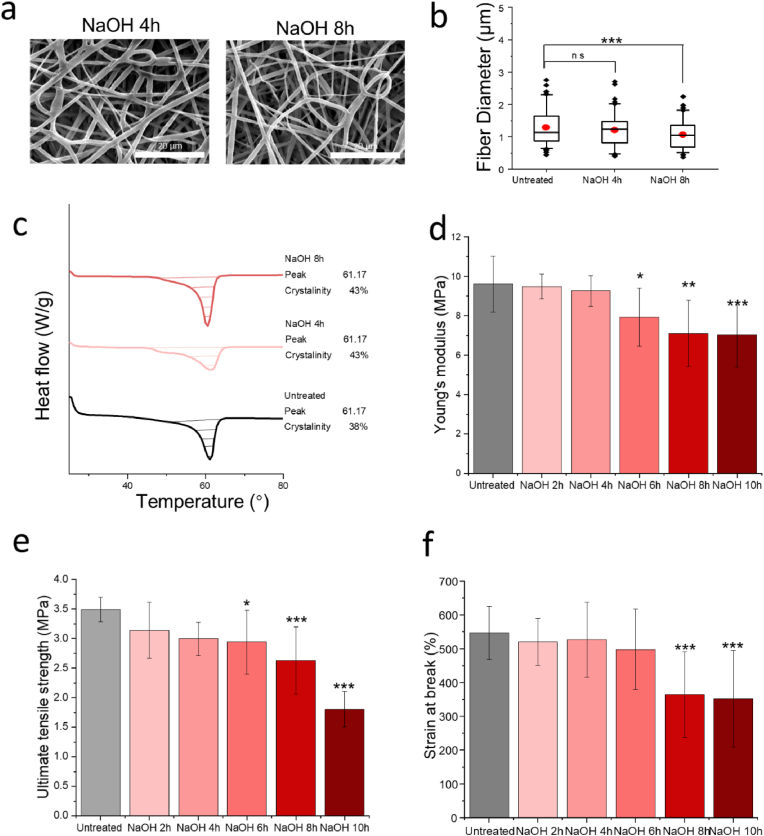
The physical changes of 2D PCL fibres under 1 M NaOH solution treatment after 2, 4, 6, 8, and 10 h (h). a) SEM images of PCL fibres treated with 1 M NaOH solution for 4 and 8 h. b) Comparison of fibre diameters. c) Differential scanning calorimetry (DSC) thermogram of PCL fibres treated with 1 M NaOH solution for 4 and 8 h. d) Young's modulus, e) ultimate tensile strength, and f) strain at break of PCL fibres treated at each time point. Statistical significance is indicated by P-values, where ∗, ∗∗, and ∗∗∗ correspond to P < 0.05, P < 0.01, and P < 0.005, respectively, with comparisons made between the untreated group and NaOH-treated groups at each time point.

The physical property changes of PCL fibres treated in 1 M NaOH solutions at 25 °C at various time points are shown in Fig. 3. The changes in morphology were analysed using SEM images (Fig. 3a). After treatment, the fibres maintained a smooth topography but appeared thinner, indicating that the NaOH solution etched the PCL fibre surface [39]. For example, a slight decrease in fibre diameter was recorded after 4 h of treatment, with the diameter measuring 1.20 ± 0.48 μm, and 8 h of NaOH processing reduced the average fibre diameter to 1.07 ± 0.40 μm (Fig. 3b).

The DSC thermogram shows an increase in crystallinity (Fig. 3c) that etching was faster in the amorphous regions compared to the crystalline regions. The peak width increased, indicating breaks in the carbon-carbon backbone chain of PCL in the crystalline area [39]. The peak location remained the same as in the untreated group at 61.17 °C. The duration of sodium hydroxide treatment adversely affects the mechanical properties of PCL fibres (Fig. 3d, e and f). The untreated electrospun samples measured 9.62 ± 1.42 MPa in Young's modulus, 3.49 ± 0.21 MPa in maximum strength, and 272.69 ± 42.50 % in strain at break. Young's modulus and ultimate tensile strength decreased slightly during NaOH treatment. A significant reduction in mechanical properties was observed in the strain at break at the 8-h treatment time point.

The amorphous regions in PCL contribute to the maximum strain, while the crystalline regions contribute to the ultimate tensile strength [39]. Therefore, the reduction in these mechanical properties indicates that continuous NaOH etching gradually affects the crystalline regions [39], with 4 h of treatment sufficient to begin etching the crystalline regions, causing structural weakening. After 8 h, the amorphous regions experienced significant damage, leading to a notable decrease in overall mechanical integrity.

**Fig. 4:**
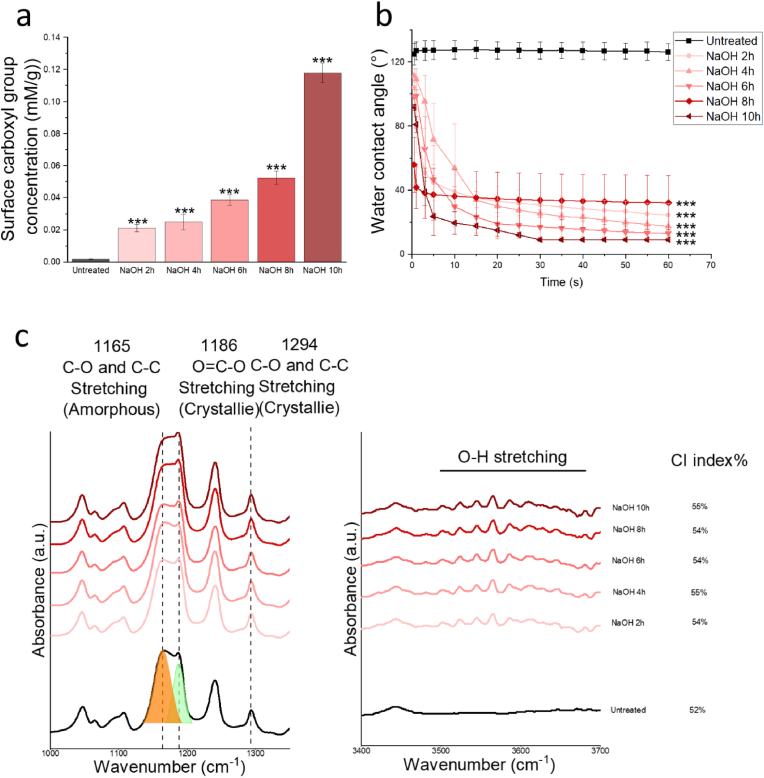
The chemical changes of 2D PCL fibres under 1 M NaOH solution treatment after 2, 4, 6, 8, and 10 h (h). a) The carboxyl group density on untreated and NaOH-treated electrospun PCL fibres. b) Wettability changes. c) FTIR spectrum comparison between 1000 cm^−1^ and 1400 cm^−1^ and 3400 cm^−1^ to 3700 cm^−1^. Statistical significance is indicated by P-values, where ∗, ∗∗, and ∗∗∗ correspond to P < 0.05, P < 0.01, and P < 0.005, respectively, with comparisons made between the untreated group and NaOH-treated groups at each time point.

The surface carboxyl groups were measured using TBO colorimetry, where TBO non-specifically adsorbs to carboxyl groups (Fig. 4a). The untreated group showed 0.0016 mM/g carboxyl groups on the surface. NaOH treatment demonstrated an increasing trend in exposed carboxyl groups on the surface, with a remarkable increase in all NaOH-treated groups: 0.024 ± 0.005 mM/g after 4 h of treatment and 0.052 ± 0.003 mM/g after 8 h. Wettability significantly improved via NaOH treatment, with all treatment groups showing quick water droplet absorption within 1 min, while untreated PCL fibres showed a stable water contact angle of around 125° (Fig. 4b). This improvement in wettability aligns with the increasing carboxyl groups on the PCL fibre surface.

The FTIR spectrum emphasized the ranges between 1000 cm^−1^ to 1400 cm^−1^ and 3400 cm^−1^ to 3700 cm^−1^ (Fig. 4c). The peak cluster in the first range represents covalent bonds between carbons and oxygens, in both amorphous and crystalline states [40,41]. The shape of the peak around 1180 cm^−1^ slightly changed during treatment. This peak can be fitted into two peaks at 1186 cm^−1^ and 1165 cm^−1^, attributed to the changes in O=C-O stretching in crystalline states and the mixture of C-O and C-C stretching from the amorphous area [[40], [41], [42]]. In general, as the treatments progressed, the peak at 1165 cm^−1^ decreased, indicating that surface modifications preferentially occurred in the amorphous area. This is consistent with the crystallization index (CI) [[43], [44], [45]], which justifies the changes in the relative intensity of the peaks at 1294 cm^−1^ and 1165 cm^−1^, showing the shifts in C-O and C-C bonds from amorphous and crystalline areas, respectively. The CI values aligned with the DSC results. For the range between 3400 cm^−1^ and 3700 cm^−1^, a broad peak was recorded in the untreated group, attributed to O-H stretching [46,47]. The increased peak strength indicates the breakage of the ester group and the introduction of hydroxyl groups [[43], [44], [45]].

### 3.3 Gelatin conjugation

**Fig. 5:**
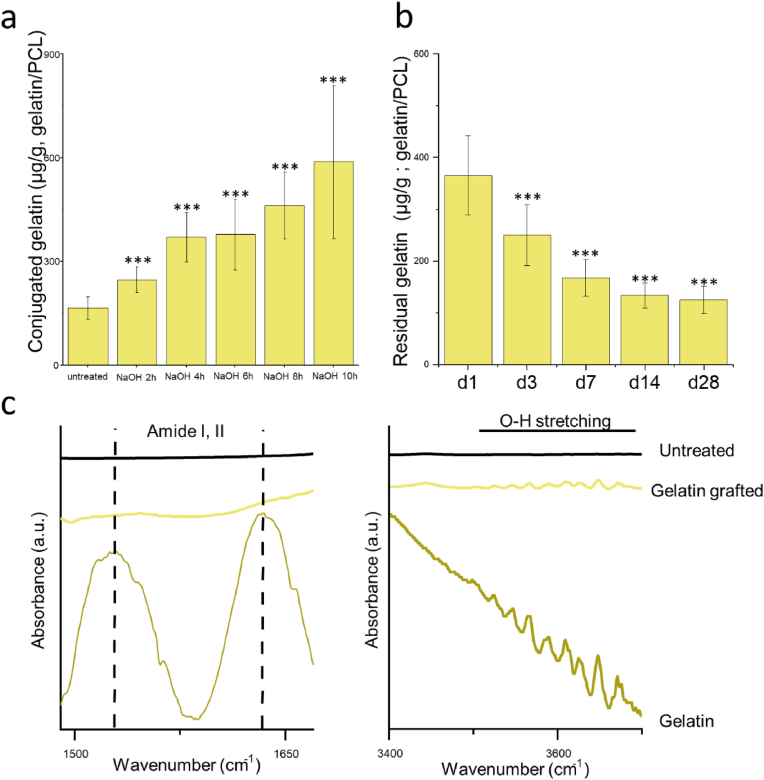
Gelatin conjugation and degradation on 2D PCL fibres (units: amount of gelatin per gram of PCL). a) The density of gelatin conjugated onto PCL following various treatment durations. b) Four-week degradation profile of the conjugated gelatin, indicating its concentration on the PCL. c) FTIR spectrum analysis of gelatin conjugation, shows the full spectrum of gelatin derived from cold fish water. The figure also compares untreated PCL with gelatin-conjugated PCL, focusing on the regions between 1450 and 1700 cm^−1^ for amide bands and between 3400 and 3700 cm^−1^ for OH stretching. Statistical significance is indicated by P-values, where ∗, ∗∗, and ∗∗∗ correspond to P < 0.05, P < 0.01, and P < 0.005, respectively. The comparisons were made between the untreated group and NaOH-treated groups at each time point for a). The residual gelatin content at each time point was compared to the initial gelatin amount on Day 1 for b).

The amount of conjugated gelatin on untreated and NaOH-treated PCL fibres at different timepoints was further measured (Fig. 5a). Gelatin was conjugated via the NHS/EDC method. The untreated PCL showed 177 ± 17 μg of gelatin conjugated per gram of PCL. After 4 h of NaOH treatment, the gelatin amount significantly increased to 350 ± 60 μg, with a slight increase at 6 and 8 h, followed by another significant increase at 10 h. Although the carboxyl density increased significantly at 4, 6, and 8 h (Fig. 4a), this increase did not correspond to a similar increase in the gelatin density (Fig. 5a), which showed only a slight rise. This discrepancy could be attributed to the possible situation that as carboxyl groups increase, the efficiency of gelatin conjugation could be limited by steric hindrance or the accessibility of the conjugation sites, leading to a slower increase in gelatin density compared to the rapid increase in carboxyl density [48]. Considering the above, the 4-h NaOH treatment at 37 °C was selected for PCL treatment before gelatin conjugation.

For gelatin-conjugated samples treated with NaOH for 4 h, gelatin degradation was researched over 28 days (Fig. 5b). The conjugated gelatin amount gradually decreased, with 133.26 ± 24.41 μg of gelatin found on 1 g of PCL at 28 days, which is around one-third of the gelatin compared to day 1, indicating the stability of gelatin conjugation. The conjugated gelatin was characterized using FTIR (Fig. 5c). The full spectrum of gelatin was revealed via FTIR, showing Amide I, Amide II [49], and OH stretching broad peaks. These peaks were also recorded in the gelatin-conjugated PCL.

**Fig. 6:**
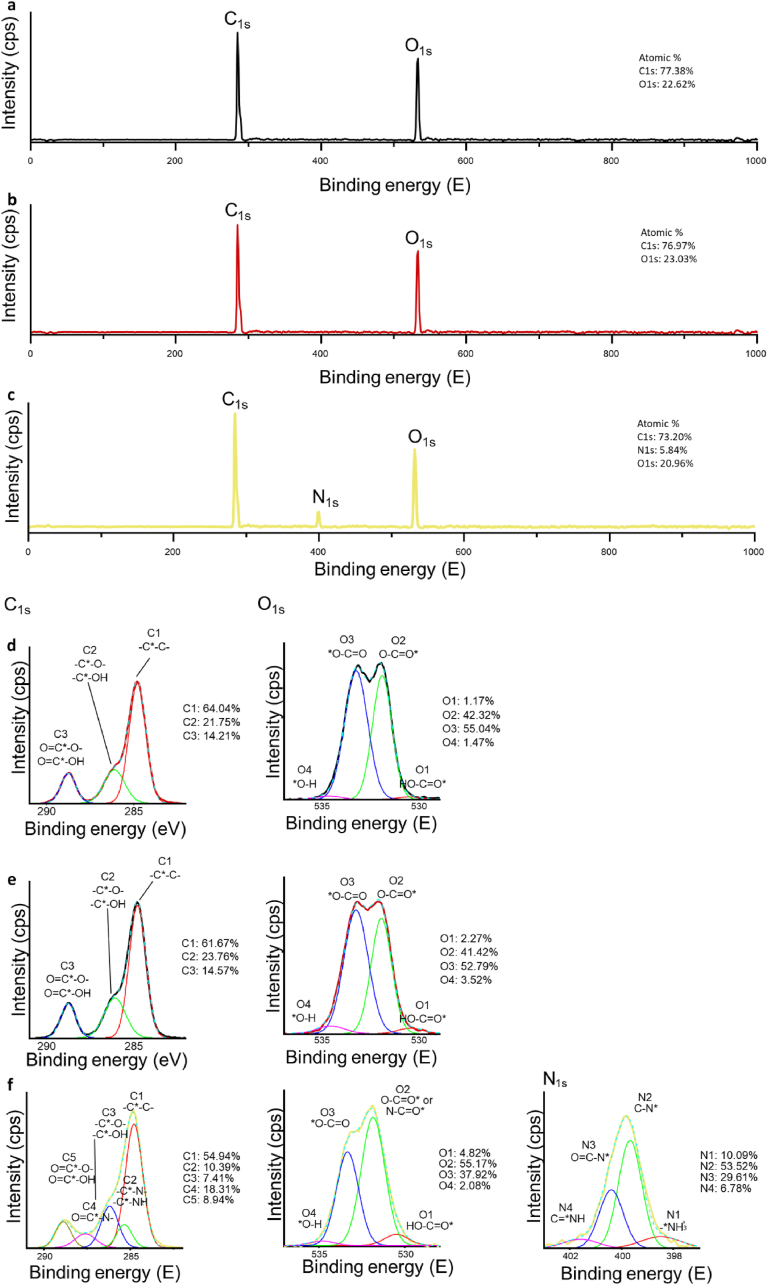
XPS analysis of gelatin conjugation presents the full XPS spectrum along with individual element spectra for C1s and O1s. The spectra are shown for a, d) untreated PCL, b, e) PCL treated with NaOH for 4 h, and c, f) gelatin-conjugated PCL. For the gelatin-conjugated PCL, the individual element spectra for N1s were also measured.

Finally, the XPS data of C1s, O1s, and N1s were measured (Fig. 6). In the C1s spectrum, which was fitted into three peaks, changes are represented in -C-C-, the mixture of -C∗-O-, -C∗-OH, and the combination of O=C∗-O- and O=C∗-O-H [50,51]. The ratios of C1, C2, and C3 indicate the breaking of the ester group and the introduction of a carboxyl group, which is consistent with the O1s spectra. In the O1s spectrum, a slight increase in the peak corresponding to -O-H groups was observed, indicating the introduction of hydroxyl and carbonyl groups. For the N1s spectrum, peaks corresponding to the -O=C-N group were detected, indicating the crosslinking between the carboxyl and amino groups [52,53].

### 3.4 In vitro evaluation

**Fig. 7:**
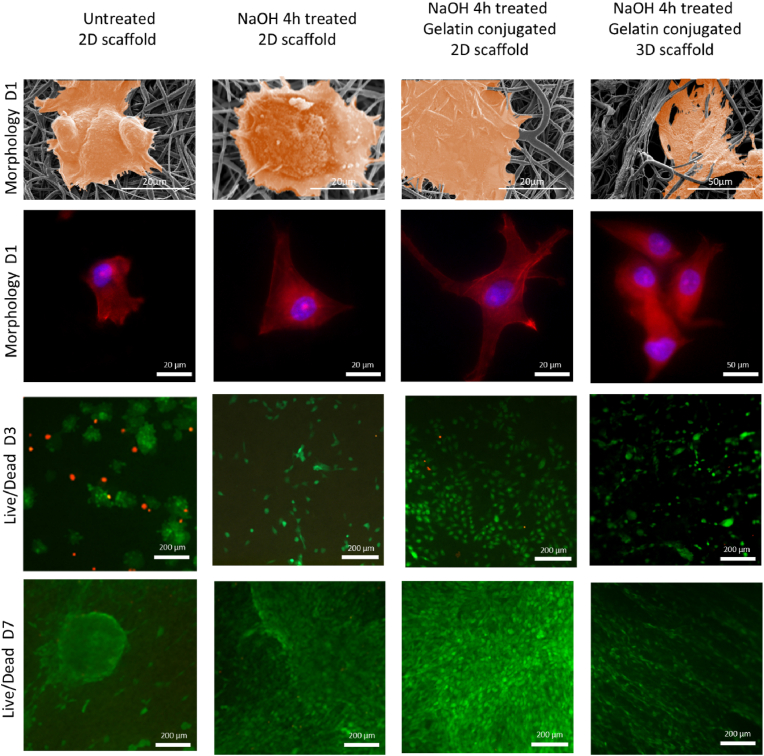
Cell Morphology, cytoskeleton, and viability. Cell morphology 24 h post-seeding on untreated 2D PCL scaffold, NaOH-treated 2D PCL scaffold, gelatin-conjugated 2D PCL scaffold, and gelatin-conjugated 3D PCL scaffold are displayed. Cytoskeletal structures are stained with phalloidin and appear in red, while nuclei are stained with DAPI and appear in blue. Cell viability is assessed using the Live/Dead kit, with live cells appearing green and dead cells appearing red, at both 3-day and 7-day time points.

To evaluate the effects of gelatin conjugation on the behaviour of bone marrow mesenchymal stem cells (BMSCs) with 2D and 3D scaffolds, the cell-scaffold interface was observed on Day 1 (D1) by Scanning Electron Microscopy (SEM) examination, and the cell cytoskeleton was imaged via phalloidin staining. Live/Dead dye-stained cells were observed via a fluorescence microscope to evaluate cell viability (Fig. 7). It was observed that BMSCs attached to all specimen groups after 24 h of culture.

In the untreated group, cells exhibited an irregular shape, with visible filopodia attempting to spread and attach to the fibres. The cell size on NaOH-treated specimens was significantly larger than the pristine specimens, as shown by the phalloidin staining examination. The cells on treated specimens have a higher number of filopodia, and these filopodia adhere to the nanofibre, especially on gelatin conjugated nanofibres, and extend along the fibre to super filopodia with lengths greater than 5 μm. The formation of filopodia can directly impact cytoskeleton tension, thus directly affecting mechanotransducive pathways. Filopodia maturation levels are thus important to cell adhesion to the fibre surface and subsequent cellular response. SEM examinations also revealed that cell cytoskeletal features on pristine specimens were different from that on treatment specimens. Cells on pristine specimens were “clump” and poorly spread, and nucleolus appeared thicker, but flattened in the peripheral regions. The cell membrane was also shown limited protrusions. In comparison, the cell membrane on treated specimens, especially gelatin-conjugated specimens, exhibited thin and fully flattened morphology indicating the treatment enhanced the cells attachments to the nanofibre surfaces. Furthermore, in the gelatin-conjugated 3D porous scaffold, the BMSCs formed cellular bridges across interconnected loose yarn and spread well into the deeper fibre layers, indicating infiltration, as observed in SEM examinations (Fig. 7).

Phalloidin staining revealed distinct actin filament organisation in different groups. In the untreated group, the actin filaments were sparse and disorganised. In contrast, the NaOH-treated group showed extended cytoskeleton organisation with well-polarized actin filaments. The NaOH/gelatin-treated group exhibited a more extensive and organised actin network, indicating robust cytoskeletal integrity and cell spreading.

For Live/Dead testing, a significant increase in the number of BMSCs was observed over 7 days on all scaffolds. Cells were uniformly distributed on NaOH/gelatin-treated samples on both D3 and D7. Meanwhile, cells covered most of the sample surfaces on both untreated and NaOH-treated scaffolds. More obvious aggregation was observed on untreated scaffolds, which could be attributed to undesirable BMSCs adhesion, consistent with SEM images. One possible reason for this aggregation could be that BMSCs prefer to adhere to other BMSCs rather than PCL, indicating undesirable bioactivity. Due to the undesirable bioactive surface, the untreated scaffold exhibited a higher number of dead cells on D3.

**Fig. 8:**
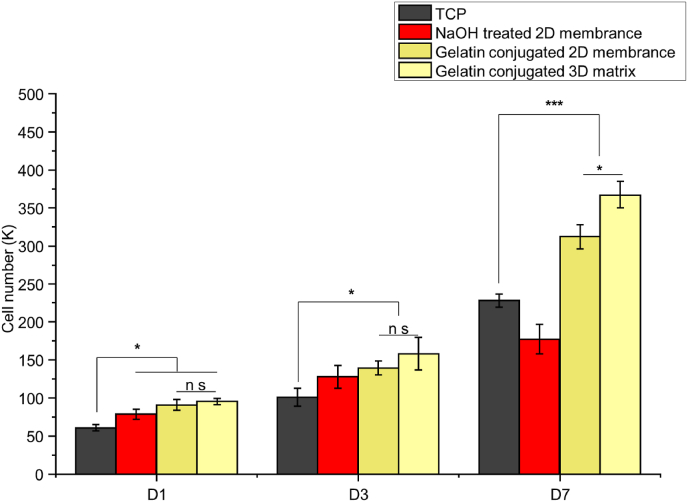
Measurement of cell proliferation using PrestoBlue assay at Days 1, 3, and 7 on tissue culture plate (TCP), NaOH-treated 2D membrane, gelatin-conjugated 2D membrane, and gelatin-conjugated 3D matrix. Statistical significance is indicated by P-values, where ∗, ∗∗, and ∗∗∗ correspond to P < 0.05, P < 0.01, and P < 0.005, respectively, with comparisons made between the TCP and scaffolds.

Cell proliferation was measured using PrestoBlue reagents after 1, 3, and 7 days of culture on surface-treated PCL electrospun fibres. On Day 1, 61 ± 4k for BMSCs was measured on TCP (Fig. 8). Slightly higher cell numbers were observed in all surface-modified groups, suggesting enhanced initial cell adhesion and growth. Cells continued to proliferate in all groups. By Day 7, the gelatin-conjugated 3D scaffold showed significantly higher proliferation (367 ± 17k) compared to all other groups, likely due to the 3D culture environment providing more space. The gelatin-conjugated 2D scaffold had the second-highest proliferation (312 ± 16k), indicating that gelatin modification enhances scaffold bioactivity. In conclusion, the 3D gelatin-conjugated fibres provide favourable conditions for cell proliferation, demonstrating their potential for regenerative material applications.

**Fig. 9:**
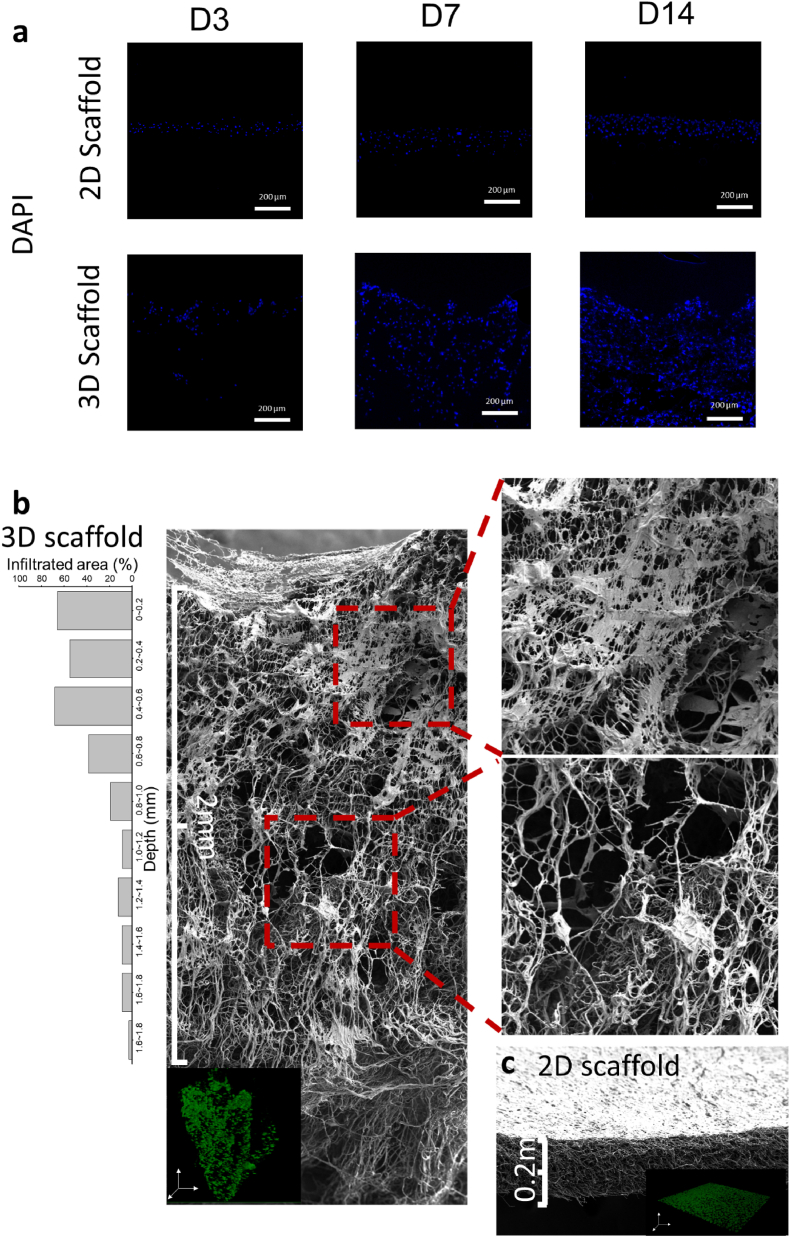
BMSCs infiltration images. a) The histological analysis of cell distribution. Cryostationed sections of gelatin-conjugated 2D and 3D scaffolds were obtained and stained at days 3, 7, and 14. Nuclei are stained with DAPI and appear in blue. SEM and confocal microscopy images of b) 3D and c) 2D scaffolds. Cross-sectional SEM images and confocal images of both 3D and 2D scaffolds after 7 days of cell seeding are shown. The percentage of the area infiltrated by cells at different depths is illustrated along with the 3D scaffolds SEM image.

Cell infiltration on the 2D dense and 3D porous scaffolds was evaluated via histology, SEM, and confocal microscopy as shown in Fig. 9. Cryosectioned slides were stained with DAPI to assess cell infiltration on 2D membranes and 3D matrices at days 3, 7, and 14 (Fig. 9a). The fluorescent images revealed the progression of cell infiltration over time. On day 3, cells attached to the surface area of the 3D samples with a depth of approximately 200 μm. Some cells even appeared in deeper regions, likely due to the large pores allowing seeded cells to fall deeper into the scaffold. By day 7, cells had migrated to a depth of around 600 μm, corroborating the SEM cross-sectional results. After 14 days of culture, cells further proliferated, leading to a higher cell density. In contrast, cells on the 2D matrix were limited to the superficial surface and showed increased density and cell layer thickness only in that area.

Four consecutive SEM images were seamlessly combined to present a comprehensive cross-section of the 3D porous scaffold (Fig. 9b) seeded with cells for 7 days, juxtaposed with the 2D membrane (Fig. 9c). Higher magnification SEM images compare cell-rich and cell-poor areas of infiltration on the 3D porous scaffold. On day 7, full-depth infiltration was observed in the 3D porous scaffold, while a confluent layer of cells was observed on the 2D dense scaffold. The cell infiltration could be attributed to the highly interconnected loose yarn, consisting of a superior porous structure. The cells migrated along the interconnected loose yarn of the 3D matrix and occupied the space between the adjacent fibres. A higher cell density was observed on the superficial layer within a 0.6 mm depth, where more than 50 % of the space was occupied by the scaffold, which aligns with the cell distribution in the confocal images where the top part exhibited a higher cell density. Notably, the fibre structure in areas with cell infiltration was markedly different from areas without infiltration. In the infiltrated regions, pores appeared more uniform and square-like in morphology, suggesting cellular remodeling of the microenvironment and potential extracellular matrix (ECM) deposition.

**Fig. 10:**
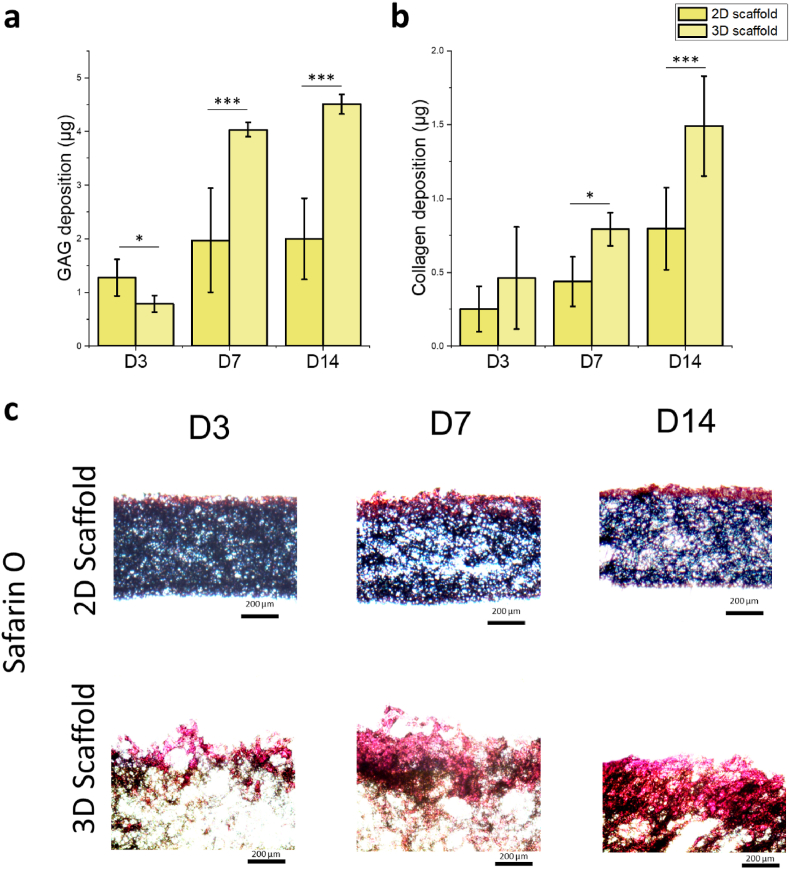
The analysis of ECM reconstruction. a) GAG and b) Collagen deposition on 2D and 3D scaffolds at days 3, 7, and 14. c) Cryostationed sections of gelatin-conjugated 2D and 3D scaffolds were obtained and stained at days 3, 7, and 14. GAGs are stained with Safranin O and appear in red. Statistical significance is indicated by P-values, where ∗, ∗∗, and ∗∗∗ correspond to P < 0.05, P < 0.01, and P < 0.005, respectively, with comparisons made between the 2D and 3D scaffolds.

ECM deposition on 2D dense and 3D porous scaffolds is shown in Fig. 10. With cell proliferation, increasing deposition of GAG and collagen was measured (Fig. 10a and b). At day 3, GAG deposition on the 2D scaffold was 1.28 ± 0.34 μg/mg, slightly higher compared to the 3D scaffold, while collagen deposition was 0.46 ± 0.35 μg/mg on the 3D scaffold, slightly higher than on the 2D scaffold. By days 7 and 14, the deposition of GAG and collagen on the 3D scaffold was significantly higher than that on the 2D scaffold. At day 14, the 3D scaffold exhibited markedly greater GAG deposition (4.51 ± 0.18 μg/mg) and collagen deposition (1.49 ± 0.34 μg/mg) compared to the 2D scaffold (GAG: 2.00 ± 0.75 μg/mg, collagen: 0.80 ± 0.28 μg/mg). These results indicate that the 3D culture environment with its porous structure promotes more substantial GAG and collagen accumulation over time. Safranin O staining images clearly show the deposition process of GAG on 2D and 3D scaffolds (Fig. 10c). The deposition of GAG aligned with the BMSC distribution, with GAG deposited on the superficial surface of the 2D dense scaffold while showing deeper deposition in the 3D scaffold.

## 4 Discussion

A major limitation of electrospun scaffolds in tissue engineering is their tendency to create a dense structure, which restricts cell infiltration. This limitation is particularly significant for zonal structure tissues such as cartilage, which require a three-dimensional (3D) environment for proper regeneration. In this study, we developed a modified wet-electrospinning technique that utilises dynamic ethanol flow and a gyrated PS foam ball to collect loose yarn, forming an interconnected high-porosity structure. This approach overcomes the constraints of conventional electrospinning by facilitating cell migration along the interconnected yarns, enabling deeper infiltration and improved tissue formation.

The innovative processing method fabricates a three-level structure with curly fibres, loose yarn consisting of curly fibres, and porous scaffold consisting of interconnected loose yarn (Fig. 2b). Deformed and curled fibres are pulled by the water flow, creating loosely porous yarns interconnected through irregularly curled fibres. Compared with static wet-electrospinning, which lacks dynamic liquid flow, the fabricated scaffold with the basic unit of the 3D structure is an interfibre mechanically locked non-woven fibre net [[54], [55], [56]]. In conventional dynamic wet-electrospinning, the yarn is the product of dynamic wet-electrospinning, but during the process of leaving the liquid bath, the internal structure of the yarn cannot remain loose due to surface tension, causing it to lose its internal pore structure and the inter-yarn connection and binding [20,57]. Full-depth infiltration benefits from the interconnected loosened yarn, as shown by cells migrating along the loosened yarn, forming cell bridges between yarns (Fig. 7), and colonising inside large pores (Fig. 9b). Compared with the conventional post-processing of the wet-electrospun yarn, such as weaving, causes the yarn to lose its universal interconnection, and cells can only migrate between the yarns via the weaving intersections, limiting the infiltration efficiency. For example, Wu's wet-electrospun silk fibroin nanoyarn scaffolds achieved 100 μm infiltration of fibroblasts at day 7 [57], and 300 μm thickness infiltration in wet-electrospun P(LLA-CL)/collagen yarn scaffolds at 10 days [20].

The rotation speed of the collection device and the concentration of ethanol in the liquid bath significantly influenced the porosity of the 3D PCL scaffold. Ethanol in the liquid bath, dynamic liquid flow, and the floating PS ball collector played key roles in the formation of the interconnected loose yarn. It is commonly accepted that lower bath liquid surface tension benefits the separation and support of the fibres by more efficiently wetting and separating deposited fibres, thereby reducing physical binding and fusion between fibres [19,58]. Ethanol, at 25 °C, with a surface tension variation from 26 mN/m to 25 mN/m between concentrations of 60 v/v% and 70 v/v%, is similar to other commonly used solvents such as methanol (60 v/v%, 30 mN/m) and propanol (1-propanol, 24 mN/m), but with lower toxicity [59]. The magnetic stir bar mounted in the liquid bath induces dynamic liquid flow, generating a complex flow field. In the central area of the liquid bath, the higher liquid speed results in greater momentum and shear forces, whereas the boundary area experiences lower speed, momentum, and shear forces. Therefore, a higher rotation speed twists and tightens the structure. For instance, Yousefzadeh et al. (2010) and Latifi et al. (2011) [60,61] reported that twisted yarns collected in the central area of the liquid bath were more tightly packed compared to those collected from the boundary area, leading to a denser structure.

Optimizing the treatment of electrospun PCL fibres requires a careful balance between conjugation efficiency and mechanical integrity. NaOH treatment cleaves ester bonds, introducing carboxyl (COOH) groups that facilitate gelatin conjugation (Fig. 4). A gentle NaOH treatment, performed similarly characterized with hydrolysis [[62], [63], [64]] while a harsh NaOH preferentially etches more in the amorphous areas, leading to slightly uneven erosion and mechanical degradation (Fig. 3, Fig. 4c). Although the density of carboxyl groups on PCL fibres increases with longer NaOH treatment time (Fig. 4a), the efficiency of gelatin conjugation does not proportionally improve (Fig. 5a). This could be attributed to a saturation point where all available conjugation sites are occupied, and additional conjugation is hindered by steric hindrance [65,66]. While longer NaOH exposures can increase conjugation efficiency due to the roughened surface topography resulting from uneven etching, considering mechanical integrity, a 4-h treatment was chosen. One limitation is that the optimisation process was conducted on 2D scaffolds, while NaOH-induced physical and microstructural changes may differ in 3D scaffolds.

Gelatin-conjugated PCL fibres exhibited significant improvements in cell behaviours compared to untreated PCL fibres. PCL consists of a long carbon-carbon backbone chain and lacks cell-recognised sequences [67]. Cell adhesion focal modulated attachment occurs via interactions with adhesion proteins adsorbed on the PCL fibre surface [68]. Although an increase in carboxyl group density makes cell adhesion easier by attracting more proteins, the conjugation of bioactive materials containing RGD sequences further facilitates cell adhesion by promoting the formation of focal adhesions and improving cell spreading [69]. These cell recognition sites on the fibres allow cells to migrate along the loosened yarn (Fig. 7) and colonize inside the pores (Fig. 8b). Gelatin conjugation was chosen using NHS/EDC due to its low toxicity [70] and stability for up to one month (Fig. 5b).

A remarkable observation was that the loosely structured fibres seemed to undergo reconstruction to accommodate the cells (Fig. 9b). Areas rich in cells showed changes in fibre arrangement, while regions lacking cells remained morphologically unchanged. This could be due to the fibres being loosely arranged and providing bonding points, making it easier for cells to reposition them. As previously discussed, the conjugated gelatin contains the RGD sequence, which cells recognise through their integrins. This led to more pronounced cell extensions, such as filopodia and lamellipodia, allowing cells to exert forces on surrounding fibres through actin polymerization and myosin motor protein activity (Fig. 7). Further, the deposition glycosaminoglycans (GAGs) seemed to rebond the fibres, aiding in fibre and ECM reconstruction.

BMSCs were chosen for this study due to their multilineage differentiation potential, particularly their ability to differentiate into chondrocytes, which is essential for cartilage tissue engineering applications [71,72]. For ECM reconstruction, 3D scaffolds exhibited significantly higher accumulation of GAGs and collagen compared to 2D scaffolds (Fig. 10a and b), which aligns with a 3D porous scaffold made of gas-foamed [73], wet-electrospun [22,74] techniques. This can be attributed to several factors. Firstly, 3D scaffolds provide a more physiologically relevant environment that facilitates natural cell-cell and cell-matrix interactions, crucial for ECM production [75,76]. In 3D environment, cells exhibit a more natural morphology and distribution, enhancing their functional and metabolic activities, thus promoting ECM component synthesis [75,76]. Moreover, a thick dense layer of cells was observed within the 3D scaffold, as confirmed by SEM and fluorescence imaging. This cellular layer may limit oxygen diffusion into deeper regions, creating a localized hypoxic microenvironment, which is less prevalent in 2D cultures [77,78]. Hypoxia induces the stabilisation and accumulation of hypoxia-inducible factor-1 alpha (HIF-1α), a key regulator of chondrocyte differentiation [79].The hypoxia-driven signalling pathway could enhance BMSCs differentiation and ECM component accumulation, particularly GAGs and collagen. Additionally, the enhanced cell infiltration in 3D scaffolds is crucial for uniform tissue formation and function [80].Thus, the combination of 3D culture environment, interconnected loosen structure and hypoxia environment improved cell infiltration in 3D scaffolds significantly boosts the production of ECM components.

Although cells infiltrated the entire matrix, ECM reconstruction primarily occurred within a 500 μm range (Fig. 10). This thickness is slightly greater than what has been reported in other studies and could be attributed to the limitations in oxygen and nutrient diffusion, which is usually around 200 μm [20,57]. Beyond this limit, cells may experience hypoxia damage, which is considered a factor in differentiation and could explain the deposition of GAGs. Additionally, cell differentiation was not defined in this study. Therefore, in future work, factors that modulate differentiation will be integrated into this scaffold to enhance ECM deposition. Specifically, depth-responsive BMSC differentiation will be modulated via smart 3D wet-electrospun freeform porous scaffolds by loading functional materials, including nano-hydroxyapatite, bone morphogenetic proteins (BMPs), and TGF-β.

## 5 Conclusion

In this study, a 3D porous wet-electrospun PCL scaffold consisting of interconnected loose yarn was fabricated, achieving full-thickness infiltration and ECM deposition. The process involved the onsite collection of loosely structured electrospun fibres. An optimized process using 65 v/v% ethanol and 100 rpm achieved a porosity of 99.5 %. Further chemical modification with sodium hydroxide (NaOH) treatment and subsequent gelatin conjugation via N-hydroxysuccinimide (NHS) crosslinking resulted in well-spread cell morphology, benefiting cell attachment and migration. In vitro evaluations revealed full-thickness cell infiltration within 7 days, as evidenced by SEM and confocal microscopy. By day 14, the scaffolds showed substantial deposition of glycosaminoglycans (GAGs) and collagen, indicating effective ECM production by the infiltrated cells. These findings suggest that this high-porosity, gelatin-conjugated 3D scaffolds offer a significant advancement in tissue engineering, particularly for applications in cartilage regeneration. The enhanced cell colonisation and matrix deposition capabilities address the limitations of traditional electrospinning techniques, facilitating more effective 3D cell culture environments. This novel approach opens new avenues for the development of functional tissue-engineered constructs and holds great promise for future clinical applications in regenerative medicine.

## CRediT authorship contribution statement

**Haoyu Wang:** Writing – review & editing, Writing – original draft, Visualization, Validation, Supervision, Software, Resources, Project administration, Methodology, Investigation, Funding acquisition, Formal analysis, Data curation, Conceptualization. **Xueying Xu:** Writing – review & editing, Data curation. **Yifei Qin:** Writing – review & editing, Data curation. **Hongyi Chen:** Writing – review & editing, Data curation. **Yuexin Wang:** Writing – review & editing, Data curation. **Joel Turner:** Writing – review & editing, Data curation. **Jianping Zhang:** Writing – review & editing. **Maryam Tamaddom:** Writing – review & editing, Writing – original draft, Supervision. **Feng-Lei Zhou:** Writing – review & editing, Supervision. **Gareth Williams:** Writing – review & editing, Resources, Project administration. **Chaozong Liu:** Writing – review & editing, Validation, Supervision, Resources, Project administration, Funding acquisition, Conceptualization.

## Declaration of competing interest

The authors declare that they have no known competing financial interests or personal relationships that could have appeared to influence the work reported in this paper.

## Data Availability

Data will be made available on request.
